# Epigenome-wide methylation differences in a group of lean and obese women – A HUNT Study

**DOI:** 10.1038/s41598-018-34003-8

**Published:** 2018-11-05

**Authors:** Kirsti Kvaløy, Christian Magnus Page, Turid Lingaas Holmen

**Affiliations:** 1 HUNT Research Centre, Department of Public Health and Nursing, Faculty of Medicine and Health Science, Norwegian University of Science and Technology, Trondheim, Norway; 2 Department of Research and Development, Levanger Hospital, Nord-Trøndelag Health Trust, Levanger, Norway; 3 Oslo Centre for Biostatistics and Epidemiology, Oslo University Hospital, Oslo, Norway; 4 Centre for fertility and health, Norwegian Institute of Public Health, Oslo, Norway

## Abstract

Knowledge of epigenetically regulated biomarkers linked to obesity development is still scarce. Improving molecular understanding of the involved factors and pathways would improve obesity phenotype characterization and reveal potentially relevant targets for obesity intervention. The Illumina Infinium HumanMethylation450 BeadChip was used in a leucocyte epigenome-wide association study (EWAS) to quantify differential DNA methylation in 60 lean compared with 60 obese young women. Replication was done in monozygotic twins discordant for obesity. At adolescence and adulthood, the two weight groups differed significantly in obesity-related traits and metabolic risk factors. Differential hypomethylation was overrepresented in obese compared to lean women. In the adjusted model, the EWAS revealed 10 differentially methylated CpG sites linked to 8 gene loci – *COX6A1P2/FGD2*, *SBNO2*, *TEX41*, *RPS6KA2*, *IGHE/IGHG1/IGHD*, *DMAP1*, *SOCS3*, and *SETBP1*– and an enhancer locus at chromosome 2 (2p25.1). The sites linked to *TEX41*, *IGHE/IGHG1/IGHD*, *DMAP1*, and *SETBP1* were novel findings, while *COX6A1P/FGD2*, *SBNO2*, *RPS6KA2*, and *SOCS3* had been identified previously with concordant direction of effects. *RPS6KA2*, *DMAP1*, and *SETBP1* were replicated in the BMI-discordant monozygotic twin cohort using the FDR of 5%.

## Introduction

Obesity has become a huge global health burden^1,2^ with the concurrent risks of co-morbidities such as cardiovascular disease, type 2 diabetes^3^, and various types of cancer^4^. The rate at which metabolic disturbances become clinically apparent in obese individuals varies and may reflect impacts by gene–environment interactions mediated by epigenetic factors. Generally, epigenetic modifications that influence early disease progression may signify mechanisms that are highly influenced by exposing factors such as nutrients^5^ and chemical components (e.g. tobacco)^6^. An obesogenic environment may comprise factors that induce inappropriate expression or silencing of genes leading to metabolic imbalances that trigger obesity development^7^. Furthermore, the stress caused by obesity may require metabolic changes to help the body to cope.

The epigenetic modification that leads to altered DNA methylation in cytosine-guanine dinucleotide (CpG) rich regions may result in altered gene expression^8,9^ with effects on timing and regulation of specific genes during various parts of the life course. Modification of gene expression through methylation is inevitably important also in obesity development. For instance, Alu elements have been shown as less methylated (hypomethylated) in overweight women and more methylated (hypermethylation) in lean and obese women^10^. In a study of monozygotic twins, 91% of the differentially methylated CpGs were hypomethylated in the obese twin compared with the discordant lean co-twin^11^. Similarly, global hypomethylation in the subcutaneous adipose tissue and leucocytes of obese individuals has been observed^12^.

In the past, elevated methylation levels have been linked to suppression of gene expression. However, this notion is oversimplified^13^, as recent research has indicated that DNA methylation at gene promoters and enhancers are associated with gene silencing, whereas higher methylation in the gene body is associated with active gene expression^14^. For obesity-related traits, two groups of epigenetic markers are hypothesized to provide effects: a more permanent group that may pre-dispose to obesity and a less permanent group that is a consequence of obesity^15^. The latter, more dynamic, group has the potential to be modified through lifestyle interventions^16^. Methylation studies of monozygotic twins have shown an association between age and increased divergence with regard to methylation, which demonstrates this mechanism’s susceptibility to environmental influence over time^17^. With regards to obesity, the authors of a previous study proposed molecular links between aging and obesity, which suggests that obesity is influenced by age-driven epigenetic changes^18^.

An intriguing question is whether DNA methylation alterations are a consequence rather than a cause of obesity, and several recent studies have indicated that the methylation alterations at the majority of BMI-associated CpGs precede obesity development^19,20^. In contrast, studies of the effects of maternal pre-pregnancy weight and/or intrauterine exposures on the offspring’s birth weight and later adiposity have supported the notion of epigenetic alterations occurring prior to obesity onset in the child. A strong association has been found between maternal pre-pregnancy BMI and decreased cord blood LEP gene methylation, which has been suggested to mediate the known association between maternal pre-pregnancy BMI and neonatal adiposity^21^. Furthermore, through a Mendelian randomization (MR) approach, it has been found that maternal glycaemia and epigenetic regulation of leptin in offspring probably contributes to long-term programming of the child’s adiposity in later life^22^.

There is still scarce knowledge of epigenetically regulated genes or biomarkers that are important in obesity development and aetiology, although epigenome-wide methylation studies have started to emerge and some recent high-powered studies have obtained some common gene findings such as *HIF3A*, *CPT1A*, and the *ABCG1* region^23–25^. Additionally, a recent EWAS identified 278 CpG sites associated with BMI distributed between 187 loci identifying genes involved in lipid and lipoprotein metabolism, substrate transport, and inflammatory pathways^20^.

Differential methylation patterns in obese individuals compared with lean individuals may reflect an unhealthy bodily state. Gaining knowledge of which molecular factors and pathways are involved in obesity development would increase our understanding of the obesity phenotype, and potentially improve our ability to assess risk factors. The aim of the study on which this article is based was therefore to identify genes and molecular pathways related to obesity by comparing epigenome-wide methylation in 60 obese and 60 lean young women.

## Methods

### Subjects and phenotypic measurements

Participants (age range: 23–31 years) were selected from non-pregnant women who had participated in the third wave of the HUNT Study^26^, HUNT3 (2006–2008). In addition to being a cross-sectional survey, HUNT3 was also a follow-up of previous adult surveys, HUNT1 (1984–1986) and HUNT2 (1995–1997), as well as of the adolescent survey, Young-HUNT1 (1995–1997)^27^. Of the 8983 individuals who participated in Young-HUNT1, only 1801 (788 males and 1013 females) also participated and provided DNA as adults in HUNT3^28^. Since there were more female than male participants in this sample and we wanted only to include one gender, our study sample consisted of the 60 women with highest BMI (median: 37.04 (32.3; 54.3)) and the 60 with the lowest BMI (median: 18.88 (16.3; 21.2)), selection based on the adult age based BMI z-scores. Data on parental BMI was also available to us, which enabled weight correlations.

Trained nurses or technicians used standardized weight scales and meter bands to measure participants’ weight, height, and waist circumference (WC). Height was measured to the nearest centimetre (cm) and weight to the nearest 0.5 kilogram (kg). WC was measured to the nearest centimetre by applying a non-stretchable band horizontally. BMI was calculated as weight in kg/height in m^2^. The BMI z-score indicates the standard deviations (SD) of the obesity measure either above (positive values) or below (negative values) the expected mean. Blood pressure, total cholesterol, high density lipoprotein cholesterol, blood glucose, and triglycerides were measured in the same way as described by van Vliet-Ostaptchouk *et al*.^29^. Numbers of smokers were more or less equally distributed in the two groups (13 in the lean and 16 in the obese group). Education (two-level categorization based on the Norwegian Standard Classification of Education (NUS2000) was recorded as either low = 0–13 years school attendance or high ≥ 14 years school attendance^30^.

### Infinium HumanMethylation450 BeadChip data acquisition and processing

DNA was extracted manually from the buffy coat fractionated from EDTA whole blood using the Gentra Purgene blood kit (QIAGEN Science, MD, USA). DNA samples were quantified using both NanoDrop Spectrophotometer (Thermo Scientific, Wilmington, DE, USA) and PicoGreen DNA methods. Samples (750 ng) were bisulfite converted using the EZ DNA Methylation Kit (Zymo Research, CA, USA). DNA samples were hybridized to the BeadChip arrays by the Genomics Core Facility (GCF) at the Norwegian University of Science and Technology (NTNU), Trondheim, Norway. HumanMethylation450 BeadChips (Illumina, San Diego, CA) were processed according to the manufacturer’s instructions. The BeadChip interrogates 485,000 methylation sites at single-nucleotide resolution. Annotations were done using the UCSC Genome Browser on Human Feb. 2009 (GRCh37/hg19) Assembly. BeadChip batch effects were present, and illustrated using PCA (see Supplementary Fig. 1), although not influenced by BMI since plate location of individuals with high or low BMI was unknown to the laboratory personnel.

### Statistical analysis

The methylation data were loaded into R using the *minfi* pipeline^31^. Probes with bad detection values (detection P > 0.8) were removed, as well as any cross-reactive probes^32^ and probes targeting the sex chromosomes. Control normalization was performed and the Type I and Type II probes were normalized together using the beta mixture quantile normalization pipeline in the R package wateRmelon^33^. To avoid influence from extreme values, the methylation data were trimmed by removing values that were outside three times the interquartile range from the mean. The methylation data was visually inspected using the first principal components to identify potential batch effects or genetic outliers. The beta values were then transformed to M-values using a logit_2_ transform to minimize the heteroscedasticity in the statistical models. The identified methylation differences indicated the amount and direction of effects between the obese group and the lean group. Negative values denoted hypomethylation in the obese cases, and positive values hypermethylation. The cell type composition (components: CD4^+^T cells, CD8^+^T cell, NK cells, B cells, monocytes, and granulocytes) was estimated using Houseman estimates^34^ and the reference set from Reinius *et al*.^35^. This was added as any other confounder or covariate. Two models were tested, in both cases with the BMI-based weight group categorization (0/1) as the exposure and the individual CpGs as the outcome, using robust linear estimation in R^36^. For Model 1, no adjustment variables were included. Model 2, same as Model 1, but adjusted for smoking, batch as fixed effects, and estimated cell type composition. The p-values were then subjected to multiple testing penalties, using the false discovery rate (FDR) of 5%.

Differential CpG sites identified with p-values < 0.05 were compared with obesity susceptibility loci reported in large GWAS with robust p-values (e.g.^37^). Further, dissimilarities between the two weight groups concerning means of anthropometric and metabolic risk measures were tested using the Mann-Whitney U test (SPSS, version 20). Differences in education level were tested by Pearson’s chi-squared test.

CpG sites associated with the BMI-based weight groups in the discovery sample after multiple testing penalties were tested in a replication sample of 30 BMI-discordant monozygotic twins^11^, using robust linear regression with BMI (exposure) coded as 0/1. In the replication data, no covariates other than sex were available, making this comparable with Model 1.

### Ethics approval and consent to participate

All participants gave a written informed consent. The protocol was in accordance with the Helsinki Declaration and approved by the Regional Committee for Ethics in Medical Research and the Norwegian Data Protection Authority. Register number for the HUNT3 Survey at the Norwegian Regional Committee for Ethics in Medical Research: 4.2006.250, dated 6 April 2006.

## Results

### Study subjects

The 60 obese and 60 lean women (mean age 27.2 years) included in the study had participated both as adolescents in the Young-HUNT1 Survey (1995–1997)^27^ and as adults in the HUNT3 Survey (2006–2008)^38^. The main characteristics of the women at both time points are summarized in Table 1. The two weight groups were selected based on their adult age-adjusted BMI z-score estimates from 1805 individuals described elsewhere^28^. The median BMI in the obese and lean groups was 37.04 (95% CI: 32.3; 54.3) and 18.88 (95% CI: 16.3; 21.2) respectively. The two groups were significantly different for most relevant obesity and metabolic risk factors: triglycerides, glucose, HDL cholesterol, blood pressure, serum micro C-reactive protein, thyroid stimulating hormone, and Type 2 diabetes risk score^39^ at adulthood. They also differed significantly at adolescence with regard to BMI, waist circumference, waist–hip ratio, and systolic and diastolic blood pressure. Weight data from parents of the 120 study participants who themselves had participated in the HUNT Study reflected the same directed differential weight as their offspring at three different time points within a time span of 20 years (Supplementary Fig. 2).

**Table 1:** Descriptive characteristics.

Age period	Characteristics	Lean		Obese		P^a^
		N	Median (95%CI)	N	Median (95%CI)	
Adulthood	Age (year)	60	27.17 (23; 31)	60	27.18 (24; 31)	—
	BMI (kg/m^2^)	60	18.88 (16.3; 21.2)	60	37.04 (32.3; 54.3)	<0.001
	Waist circum. (cm)	60	70.13 (59; 86)	60	112.32 (98; 139)	<0.001
	Waist-hip ratio (cm/cm)	60	0.78 (0.64; 0.91)	60	0.92 (0.78; 1.03)	<0.001
	zBMI	60	−1.34 (−1.86; −1.13)	60	2.48 (1.72; 5.31)	<0.001
	Triglyceride	60	0.92 (0.3; 2.6)	60	1.81 (0.4; 4.5)	<0.001
	Tot Cholesterol	58	4.51 (3.1; 6.0)	59	5.16 (3.4; 8.5)	0.001
	Glucose	58	5.03 (2.8; 27.3)	59	5.46 (3.9; 16.2)	<0.001
	HDL cholesterol	58	1.50 (0.9; 2.1)	59	1.16 (0.6; 2.6)	<0.001
	Syst. blood pressure	60	111.20 (87; 132)	60	123.03 (103; 147)	<0.001
	Dia. blood pressure	60	63.30 (43; 82)	60	69.85 (54; 94)	<0.001
	Type 2 diabetes risk score^b^	60	2.77 (0; 10)	60	10.72 (7; 20)	<0.001
	Serum micro C-reactive protein	50	1.71 (0.1; 39.7)	46	5.98 (0.6; 21.3)	<0.001
	Thyroid stimulating hormone	58	1.15 (0.5; 2.4)	58	1.84 (0.5; 6.5)	<0.001
	Pulse^***^	52	73.53 (53.5; 96.5)	51	78.75 (52.0; 107.0)	0.019
	Arterial pressure	52	79.52 (60; 104)	51	88.22 (70; 108)	<0.001
	Education low/high^d^	51	24/27	53	30/23	0.330^e^
Adolescence	Age (year)	60	15.94 (13; 20)	60	15.9 (13; 20)	—
	zBMI	56	−0.93 (−2.3; 0.4)	58	1.51 (−1.0; 6.4)	<0.001
	zWC	55	−0.73 (−2.0; 1.2)	58	1.31 (−1.6; 5.1)	<0.001
	zWHR	55	−0.22 (−2.2;1.8)	58	0.64 (−1.4; 3.0)	<0.001
	Syst. blood pressure	56	117.39 (100.0; 151.0)	58	125.46 (109.0; 151.5)	<0.001
	Dia. blood pressure	56	61.54 (41.5; 79.5)	58	65.40 (42.5; 80.5)	0.024
	Pulse^c^	56	77.05 (51.0; 122.5)	58	78.90 (52.0; 103.5)	0.288

^a^P-value asymptotic. Sig. (2-tailed) deduced from the Mann-Whitney U-test. ^b^Finnish Type 2 diabetes risk score^33^. ^c^Mean of second and third measurement. ^d^Low = 0–13 years school attendance, High > 14 years school attendance.

### Differentially methylation CpG sites

**Figure 1:**
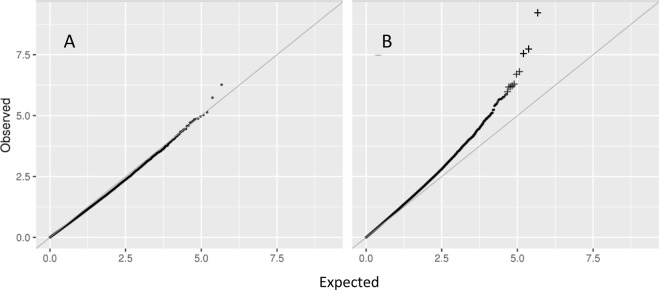
QQ plot of differential methylation sites subjected to crude analysis (**A**) and in analysis adjusted with fixed batch effects, smoking, and cell composition (**B**). Significant sites after FDR-adjustment is marked by **+**.

The epigenome-wide differential methylation analysis revealed 26982 CpG sites that differed between obese and lean individuals with nominally significant p-values (p ≤ 0.05). Of these, 10 CpG sites were significant after false discovery rate (FDR) corrections in the adjusted model (adjustments for fixed batch effects, smoking, and cell composition), as shown in Table 2 and Fig. 1. Of the 10 significant CpG sites, 9 were distributed within or near the following 8 gene loci: *COX6A1P2/FGD2*, *SBNO2*, *TEX41*, *RPS6KA2*, *IGHE/IGHG1/IGHD*, *DMAP1*, *SOCS3*, and *SETBP1*. The *SBNO2*-associated sites (cg12170787, cg18608055) and SOCS3 (cg18181703) were localized within the body of the genes. The site in *SETBP1* (cg24217948) was localized within the 5′UTR, the site near *TEX41* within an enhancer element and the *DMAP1* site (cg11683482) within a TSS1500. The *COX6A1P2/FGD2* site (cg03957124) was positioned within the south shelf of a CpG island. In addition, the significant CpG site within chromosome 2 (cg05233324) at 2p25.1 was linked to an enhancer element and hence could play a role in gene regulation (Table 2).

**Table 2:** Significant differentially methylated sites adjusted for covariates and cell composition.

CpG	Chr	Pos	Nearest gene	Effect (CI 95%)	SE	Beta values		Difference Δ	P^*^	P^**^	Relation to gene	Relation to CpG-island	Enhancer	Regulatory feature
						Low weight group	High weight group							
cg03957124§	chr6	37016869	COX6A1P2/ FGD2	−0.148 (−0.257, −0.082)	0.024	0.567	0.537	0.030	6.17 × 10^−10^	2.9 × 10^−4^		S-Shelf		unclassified
cg12170787	chr19	1130965	SBNO2	−0.160 (−0.244, −0.102)	0.028	0.594	0.566	0.029	1.93 × 10^−8^	4.4 × 10^−3^	Body			Promoter associated
cg18608055§	chr19	1130866	SBNO2	−0.210 (−0.336, −0.122)	0.038	0.600	0.564	0.036	2.81 × 10^−8^	4.4 × 10^−3^	Body			Promoter associated
cg00452308	chr2	145633791	TEX41	0.918 (0.382, 1.177)	0.175	0.986	0.991	−0.005	1.57 × 10^−7^	1.8 × 10^−2^			x	
cg05233324	chr2	8628196	—	−0.228 (−0.311, −0.115)	0.044	0.374	0.340	0.034	2.00 × 10^−7^	1.9 × 10^−2^			x	unclassified
cg17501210§#	chr6	166970252	RPS6KA2	−0.410 (−0.525, −0.206)	0.082	0.811	0.769	0.042	5.08 × 10^−7^	3.5 × 10^−2^	Body			
cg13074055	chr14	106329206	IGHE/IGHG1/IGHD	−0.482 (−0.650, −0.262)	0.096	0.558	0.487	0.071	5.95 × 10^−7^	3.5 × 10^−2^				
cg11683482	chr1	44678623	DMAP1	−0.180 (−0.267, −0.089)	0.036	0.714	0.687	0.027	6.60 × 10^−7^	3.5 × 10^−2^	TSS1500	N-Shore		
cg18181703§	chr17	76354621	SOCS3	−0.245 (−0.332, −0.142)	0.049	0.508	0.469	0.039	6.67 × 10^−7^	3.5 × 10^−2^	Body	N-Shore		promoter associated
cg24217948	chr18	42261980	SETBP1	−0.311 (−0.381, −0.156)	0.064	0.777	0.746	0.032	1.06 × 10^−6^	4.9 × 10^−2^	5′UTR	S-Shore	x	

^*^Unadjusted p-values ^**^FDR adjusted p-values. ^§^CpG sites identified by Wahl *et al*.^20^ and ^#^CpG site identified by Mendelson *et al*.^19^.

The following 3 of the 10 significant CpG sites were replicated with a FDR-adjusted p-value < 0.05 (see Table 3): *RPS6KA2* (cg17501210), *DMAP1* (cg11683482), and *SETBP1* (cg24217948). Of these, *RPS6KA2* (cg17501210) and *SETBP1* (cg24217948), had the same direction of effect as in the discovery sample.

**Table 3:** Differentially methylated CpG sites in a replication sample consisting of BMI discordant monozygotic twins.

CpG	Nearest gene	Discovery stage			Replication stage			P^**^
		Effect	SE	P^*^	Effect	SE	P^*^	
cg03957124	COX6A1P2/FGD2	−0.148	0.024	6.17 × 10^−10^	0.097	0.051	0.057	0.114
cg12170787	SBNO2	−0.160	0.028	1.93 × 10^−8^	0.036	0.030	0.227	0.369
cg18608055	SBNO2	−0.210	0.038	2.81 × 10^−8^	0.137	0.061	2.4 × 10^−2^	0.060
cg00452308	TEX41	0.918	0.175	1.57 × 10^−7^	−0.078	0.137	0.568	0.631
cg05233324	–	−0.228	0.044	2.00 × 10^−7^	−0.031	0.031	0.315	0.394
cg17501210	RPS6KA2	−0.410	0.082	5.08 × 10^−7^	−0.109	0.031	4.3 × 10^−4^	0.004
cg13074055	IGHE/IGHG1/IGHD	−0.482	0.096	5.95 × 10^−7^	−0.091	0.080	0.258	0.369
cg11683482	DMAP1	−0.180	0.036	6.60 × 10^−7^	0.095	0.038	1.3 × 10^−2^	0.043
cg18181703	SOCS3	−0.245	0.049	6.67 × 10^−7^	−0.008	0.025	0.739	0.739
cg24217948	SETBP1	−0.311	0.064	1.06 × 10^−6^	−0.106	0.040	8.9 × 10^−3^	0.043

^*^Unadjusted P-values. ^**^Benjamini-Hochberg adjusted P-values. Significant differential CpG sites using an FDR of 5% are underlined.

The 50 differentially methylated CpG sites adjusted for covariates and cell composition with the lowest p-values (6.17 × 10^−10^ to 1.32 × 10^−5^) are listed in Supplementary Table 1. The direction of effects between the obese and lean groups in 46 of the 50 differentially methylated CpG sites was negative, which denoted hypomethylation in the obese cases compared with the lean cases.

### Differentially methylated CpG sites linked to obesity susceptibility loci identified by previous GWAS

We investigating whether the differentially methylated CpG sites identified in our study coincided with previously identified obesity susceptibility genes. We addressed the susceptibility genes detected by various GWAS^37,40^. Differential CpG sites linked to obesity susceptibility genes are listed in Supplementary Table 2. A number of methylation differential sites were detected in many of the genes with 26 differential CpG sites within *KCNQ1* and 24 within *RPTOR*. The following genes showed differentiation at five or more CpG sites: *KCNMA1*, *MACROD1*, *NAV1*, *CADM1*, *GALNT10*, *SMAD6*, and *RREB1* and three differential sites were identified within *FTO*, which is the obesity susceptibility gene with strongest effect in healthy individuals^37^ (Supplementary Table 2).

## Discussion

We identified significant CpG sites with aberrant DNA methylation in 60 obese young women compared with 60 lean young women through an epigenome-wide approach. Several CpG sites deviant in the obese versus the lean group within or nearby genes have previously been recognized in other epigenome-wide studies, including *COX6A1P2/FGD2*, *SBNO2*, *RPS6KA2*, and *SOCS3*. To our knowledge, significant CpG sites linked to the genes *TEX41*, *IGHE/IGHG1/IGH*, *DMAP1*, and *SETBP1* had not been identified previously as having genome-wide significance. Additionally, we detected a novel aberrant CpG site linked to an enhancer at chromosome 2 (2p25.1). We found that differentially methylated sites were more likely to show a pattern of hypomethylation (i.e. a lower degree of methylation) in the obese group compared with the lean group, which is in agreement with observations in other epigenome-wide studies addressing obesity as a target. *RPS6KA2*, *DMAP1*, and *SETBP1* were replicated in a BMI-discordant monozygotic twin cohort.

The scientific study of epigenetics is still in its infancy with regards to understanding the complexity related to environmental effects, interactions with age, and role in developmental programming. Is an epigenetic modification causative (i.e. does it occur prior to, for instance, obesity development) or is it an effect of obesity? Although it is not easy to answer this question, it is very important to address it. Recent evidence^19–22^ suggests that DNA methylation alterations are predominantly the consequence of obesity rather than the cause of it. However, the approach chosen in our study did not enable us to distinguish the direction of the causative effect.

Several of the top-hit gene findings identified in our study have previously been associated with obesity-associated traits, and the significant CpG sites within or near the genes *COX6A1P2/FGD2* (cg03957124), *SBNO2* (cg18608055), *RPS6KA2* (cg17501210), and *SOCS3* (cg18181703) were all detected in a recent EWAS of adiposity^20^. Mendelson *et al*. also identified the sites within *RPS6KA2* and *SOCS3*^19^. Four sites reported by Wahl *et al*.^20^ – cg03050965 (*S1PPR1*), cg23068772 (*CRHR2*), cg06207201 (*SNX20*), and cg06192883 (*MYO5C*) – were identified in our study too, although they did not obtain significance after multiple testing. However, of all correlated sites identified, the directions of effects were in agreement with previous observations^19,20^, which is reassuring with regard to the quality of our dataset and our ability to identify sites robustly. The site cg17501210 (*RPS6KA2*), displayed the strongest effect (p-value: 4.9 × 10^−7^) in the study by Al Muftah *et al*.^41^.

The significantly differentiated CpG sites discovered in our study, but not identified in other recently published studies, were cg12170787 (*SBNO2*), cg00452308 (*TEX41*), cg13074055 (*IGHE/IGHG1/IGH*), cg11683482 (*DMAP1*), cg24217948 (*SETBP1*), and cg05233324 (2p25.1). The site cg00452308 near *TEX41* (testis expressed 41) (non-protein coding), has displayed differential methylation in growth-discordant monozygotic twins^42^, and a gain of methylation was observed in the growth-restricted twin – a finding that was supported by our results, where the site was hypomethylation in the obese group.

Of the 10 most significant differentially methylated CpG sites identified in our discovery sample, 3 were reproduced with a FDR-adjusted p-value < 0.05 in the replication sample. Of these, the effects of only the two most significant ones, *RPS6KA2* and *SETBP1*, were directionally consistent. The other markers were not directionally consistent, which could have been due to lack of statistical strength and hence too small effects obtained at the replication stage. The differences between the discovery and replication cohort might also have influenced the results. Although the age ranges were similar, the replication cohort was of Finnish origin and consisted of 17 male twins and 23 female twins^11^. In addition, we were unable to make adjustments in our replication sample, which might have precluded our ability to obtain accurate estimates.

DNA methyltransferase 1 (*DMNT1*) functions during DNA replication, copying the DNA methylation pattern from parental DNA strand onto the newly synthesized daughter strand. It interacts with *DMAP1* (*DNMT1*-associated protein), which has an intrinsic repressive activity and helps to maintain DNA methylation in a heritable manner^43^. In previous studies, proteins involved in DNA methyl transfer have not been very strongly associated with obesity, although some studies have indirectly suggested such a role. Increased expression of DNA methyltransferase 3a *Dnmt3a* in the adipose tissue of transgenic mice suggests that it contributes to obesity-related inflammation^44^. In chickens, reduced expression of *DNMT1* and *DMAP1* has been proposed as one of the adaptive mechanisms to chronic early-life nutritional stress^45^ and thereby indirectly linked to weight regulating processes. In women, *DMAP1* has been shown as downregulated and differentially methylated in adipose tissue of individuals with polycystic ovary syndrome^46^ where the PCOS individuals had higher BMI and greater waist–hip ratio than did the non-PCOS individuals.

*SETBP1*, which encodes the SET-binding protein 1, seems not to have been associated with obesity previously. Its function is unknown, although it is involved in several haematological malignancies^47^, such as myeloid leukaemia development^48^, and as de novo germline mutations in the Schinzel-Giedion syndrome^49^. The SETBP1 protein possesses three conserved AT-hooks^49^, suggesting a more general role as a DNA-binding protein. It has recently been shown to form a multiprotein complex, including HCF1, KMT2A, PHF8, and PHF6, resulting in increased chromatin accessibility and transcriptional activation^47^.

*RPS6KA* (Ribosomal protein S6 kinase 2 alpha) is interesting, as three previous studies targeting BMI also have identified it to be differentially methylated with a corresponding directional effect to our study. *RPS6KA* is one of the genes where the promoter DNA methylation status is linked to insulin signalling and angiogenesis. This happens through an interplay with the cytokine IL-6 and reduced levels of the methyl transferases DNMT1 and DNMT3B^50^.

Obesity is an inflammatory predisposition associated with chronic activation of cells of the innate immune system in which B cells seem to play a major role^51^. In our study, the identified association between obesity and the hypomethylated CpG site cg13074055 near the gene cluster of immunoglobulin heavy constant genes (*IGHE/IGHG1/IGH*) is interesting in this respect, as it suggests the expression of immunoglobulins to be potentially modified. Further, five differentially methylated sites identified in our study – cg03957124 (*COX6A1P2/ FGD2*), cg18608055 (*SBNO2*), cg17501210 (*RPS6KA2*), cg18181703 (*SOCS3*), and cg06192883 (*MYO5C*) – have been associated with serum C-reactive protein (CRP) levels in a recent study^52^, confirming the link between obesity and inflammation.

The BMI-associated differential methylation sites reported here appear partly correlated with known BMI-associated GWAS loci^37,40,53–57^. Since the cumulative effects of disease-associated SNPs have not been proven to account for the majority of complex-trait heritability, epigenetics is believed to explain some of the ‘missing heritability’ because epigenetic markers’ effects on regulation also provide a functional role for some of the intergenic loci previously associated with disease^16^. Many of the differential CpG sites identified in our study coincided with CpGs within or nearby genes associated with obesity through linkage to genetic variants. This confirms their role in obesity developmental mechanisms. *KCNQ1* and *RPTOR*, which showed differences between lean and obese women at many methylated sites, have both been linked to obesity in earlier studies. *KCNQ1* has also been robustly associated with type 2 diabetes^58^, and genetic variants near *KCNQ1* have shown specific associations with risk of obesity-linked type 2 diabetes^59^. Furthermore, differential methylation has previously been detected within the *KCNQ1* gene in women both before and after gastric bypass and significant weight loss^16^. *RPTOR*, the regulatory-associated protein of mTOR, is involved in the control of the mammalian target of rapamycin complex 1 (mTORC1) activity, which regulates cell growth and survival, and autophagy in response to nutrient and hormonal signals^60^. *RPTOR* has previously been directly associated with overweight in a GWAS study^53^.

Despite lacking methylation data at several time points and for the ancestors of our study participants, we know that our two weight groups were substantially different also 11 years ahead of our study. In addition, parents of the individuals in the two weight groups were significantly different in terms of weight corresponding to the offspring weight group at three different time points with the earliest corresponding to the participants’ age range 2–9 years. This meant our participants were good candidates in terms of exposure to several obesity promoting factors that could have influenced epigenetic differential modifications over time.

Our finding that differentially hypomethylated CpGs were overrepresented in obese women supports the work of Ollikainen *et al*., who made the same finding in their obese co-twins^11^. In their twin study, the hypomethylated CpGs were most prevalent within repressed and weakly transcribed regions. Several studies related to animal models are interesting in this respect. For example, reduced hypothalamic methylation of the *POMC* and *GR* promoters that occurred as a result of undernutrition during pregnancy in sheep, suggested that nutritional programming events would increase risk of obesity and diabetes development later in life^61^. Furthermore, a study that primarily focused on drosophila demonstrated paternal diet induced effects on offspring obesity susceptibility. A paternal high-sugar diet was shown to increase gene expression preferentially of heterochromatic-embedded genes in embryos. The same study gave further evidence of similar systems regulating obesity susceptibility in mice and humans^62^. A twin study revealed evidence of transcriptome-wide de-silencing and approximately fivefold more upregulated genes versus downregulated genes in the obese co-twins^63^. Moreover, the results of recent epigenome-wide obesity studies that focused more directly on genes have confirmed that increased hypomethylation is related to obesity^19,20,41^.

Our study was restricted to the chosen extreme trait design addressing methylation differences related to general obesity through BMI measurements. Since our targeted weight groups significantly differed also in other metabolic traits such as blood lipids, glucose, and blood pressure, we could not preclude the possibility that our identified CpG sites were associated with these correlated traits. The EWAS results reported by Wahl *et al*.^20^ provide evidence that methylation changes in blood initially associated with increased BMI levels also influence future type 2 diabetes risk. Interestingly, in relation to our differentially methylated CpG findings, differential methylation of *SBNO2* was associated with age-independent cardiovascular risk in a recent study^64^. Further, the CpG site cg18181703 (*SOCS3*) identified in our study, has been associated with metabolic syndrome (MetS) traits such as central obesity, fat depots, insulin responsiveness, and plasma lipids^65^, as well as type 2 diabetes incidence^66^.

The main limitation in our study was the low number of individuals included, which challenged the statistical strength of the findings^67^. Another limitation was the use of peripheral blood, which consisted of a mixture of blood cells. However, the latter limitation might not have been very severe, as there are coherent results from comparisons of blood-based and tissue-specific methylation patterns^12^. The extreme trait design chosen enabled us to identify differential methylation sites in agreement with previously detected markers in much larger studies. According to Berndt *et al*.^53^, this type of study design may provide greater genetic contribution and enrichment for highly penetrant variants. Additionally, effect sizes may be larger and a smaller proportion of the variance may be attributable to environmental factors^53^. Our novel differentiated CpG sites linked to *DMAP1* and *SETBP1* should be further confirmed in other cohorts or by meta-analysis.

One advantage in our study was that our participants represent an ethnically homogenous population. Approximately 98% of the individuals who participated in the HUNT3 survey were of ethnic Norwegian origin^38^. Furthermore, only females were included, which avoided the risk of sex-specific differences from affecting our results. Our sample was also age-limited, which prevent age-specific methylation changes from minimizing our findings.

In our study, the identification of differentially methylated CpG sites that showed divergence in young women was affected by their obesity and probably by an obesogenic environment since adolescence and childhood compared with their corresponding lean peers could further our understanding of the role of epigenetics in obesity development. Although several of the identified top hits were in agreement with findings from previous studies, the results needs further verification and replication.

## Conclusion

Comparative analyses of genome-wide leukocyte DNA methylation variation in 60 obese young women compared with 60 lean young women revealed differential methylation in several CpG sites, with overrepresentation of hypomethylation in the obese group. We found 10 significant differentially methylated CpG sites linked to 8 gene loci – *COX6A1P2/FGD2*, *SBNO2*, *TEX41*, *RPS6KA2*, *IGHE/IGHG1/IGHD*, *DMAP1*, *SOCS3*, and *SETBP1* – as well as a novel site linked to an enhancer within chromosome 2. The sites linked to the *DMAP1*, *SETBP1*, *TEX41*, and *IGHE/IGHG1/IGHD* loci were novel findings, while *COX6A1P*, *SBNO2*, *RPS6KA2*, and *SOCS3* had been robustly identified previously. The consistent replication of earlier findings strengthened our novel findings and our study provided knowledge of new molecular markers of obesity.

## Electronic supplementary material

Supplementary information

## Data Availability

The Nord-Trøndelag Health Study (HUNT) invited persons above 13 years of age and living in the county of Nord-Trøndelag to participate. The data are stored in HUNT databank and biological material in HUNT biobank. The HUNT Research Centre has been given concession to store and handle these data by the Norwegian Data Protection Authority. The key identification in the database is the personal identification number assigned to all Norwegians at birth or immigration, whereas anonymized versions of the data are sent to researchers. Due to confidentiality, HUNT Research Centre wants to limit storage of data outside the HUNT databank, and restrictions are imposed on researchers handling HUNT data files. We have precise information on all data exported to different projects and there are no restrictions regarding data export given approval of applications to the HUNT Research Centre (http://www.ntnu.edu/hunt/data).
