# Supplementary material for: Epigenome-wide methylation differences in a group of lean and obese women – A HUNT Study
Source: Sci Rep. 2018 Nov 5;8:16330. doi: 10.1038/s41598-018-34003-8 (PMC6218540; doi:10.1038/s41598-018-34003-8)
Supplement: Supplementary file 1 — Supplementary information [file 41598_2018_34003_MOESM1_ESM.pdf]

Epigenome-wide methylation differences in a group of lean and obese women – A HUNT Study

Kirsti Kvaløy[1,2], Christian Magnus Page[3,4], Turid Lingaas Holmen[1]

[1]HUNT Research Centre, Department of Public Health and Nursing, Faculty of Medicine and Health Science, Norwegian University of Science and Technology, Trondheim, Norway.

[2]Department of Research and Development, Levanger Hospital, Nord-Trøndelag Health Trust, Levanger, Norway.

[3]Oslo Centre for Biostatistics and Epidemiology, Oslo University Hospital, Oslo, Norway

[4]Centre for fertility and health, Norwegian Institute of Public Health, Oslo, Norway.

Corresponding author:

Kirsti Kvaløy

E-mail: kirsti.kvaloy@ntnu.no

Supplementary information

Supplementary table 1. The 50 most significant differentially methylated sites adjusted for covariates and cell composition.

CpG	Chr	Position	Nearest gene	Effect (SE)	P	P*	P**	Relation to gene	Enhancer	Regulatory feature group
**cg03957124**	**chr6**	**37016869**	**COX6A1P2/FGD2**	-0.148 (0.024)	6.17×10^{-10}	1.48x10-2	8.4x10-8			unclassified
cg12170787	chr19	1130965	SBNO2	-0.160 (0.028)	1.93×10^{-8}	1.41x10-2		Body		promoter associated
**cg18608055**	**chr19**	**1130866**	**SBNO2**	-0.210 (0.038)	2.81×10^{-8}	3.50x10-3	1.2 x10-9	Body		promoter associated
cg00452308	chr2	145633791	TEX41	0.918 (0.175)	1.57×10^{-7}	2.66x10-1			x	
cg05233324	chr2	8628196		-0.228 (0.044)	2.00×10^{-7}	2.45x10-2			x	unclassified
**cg17501210**	**chr6**	**166970252**	**RPS6KA2**	-0.410 (0.082)	5.08×10^{-7}	4.90x10-7	1.2x10-13	Body		
cg13074055	chr14	106329206	IGHE/IGHG1/IGHD	-0.482 (0.096)	5.95×10^{-7}	2.56x10-1				
cg11683482	chr1	44678623	DMAP1	-0.180 (0.036)	6.60×10^{-7}	4.76x10-1		TSS1500		
**cg18181703**	**chr17**	**76354621**	**SOCS3**	-0.245 (0.049)	6.67×10^{-7}	3.99x10-6	3.5x10-16	Body		promoter associated
cg24217948	chr18	42261980	SETBP1	-0.311 (0.064)	1.06×10^{-6}	5.67x10-1		5'UTR	x	
cg19800913	chrX	134186228	FAM127B	-0.142 (0.029)	1.33×10^{-6}	-		TSS200		promoter associated
cg11793449	chr17	76313872		-0.115 (0.024)	1.47×10^{-6}	1.40x10-2				unclassified/cell type specific
**cg03050965**	**chr1**	**101705237**	**S1PR1**	-0.160 (0.034)	1.71×10^{-6}	9.75x10-2	2.9x10-6	Body		promoter associated
cg02447556	chr16	845011	CHTF18	-0.440 (0.092)	1.75×10^{-6}	4.28x10-1		Body		unclassified/cell type specific
cg11694510	chr11	313354	IFITM1	-0.120 (0.025)	1.95×10^{-6}	2.17x10-2		TSS1500		promoter associated/cell type specific
ch.1.839062R	chr1	25282539	RUNX3	-0.482 (0.101)	2.06×10^{-6}	-		Body		
cg02944761	chr7	32771852	MIR550-2	-0.370 (0.078)	2.11×10^{-6}	7.07x10-1		TSS1500		
**cg23068772**	**chr7**	**30737061**	**CRHR2**	-0.190 (0.040)	2.13×10^{-6}	1.30x10-1	1.3x10-7	CRHR2		
cg01428095	chr1	228134229	WNT9A	0.168 (0.035)	2.18×10^{-6}	6.74x10-1		Body		unclassified
cg06762457	chr6	149806635	ZC3H12D	-0.254 (0.054)	2.39×10^{-6}	5.95x10-2		TSS1500		promoter associated
cg02362246	chrX	12974819		-0.527 (0.112)	2.73×10^{-6}	-			x	
cg04936619	chr17	30658537	C17orf75	-0.678 (0.145)	3.05×10^{-6}	2.39x10-1		3'UTR		
cg21585138	chr3	50645106	CISH	-0.357 (0.077)	3.39×10^{-6}	1.60x10-1		Body		
cg00694302	chr12	47609865	FAM113B	-0.465 (0.100)	3.42×10^{-6}	8.41x10-1				
cg14804531	chr11	334298		-0.221 (0.048)	3.53×10^{-6}	5.7x10-3				
cg24960291	chr3	185538892	IGF2BP2	-0.170 (0.037)	3.89×10^{-6}	8.70x10-1		Body		
cg25446098	chr17	47209664	B4GALNT2	-0.323 (0.037)	3.98×10^{-6}	2.06x10-1		TSS1500;TSS200		Unclassified/cell type specific
cg08845291	chr19	46144133	EML2	-0.413 (0.091)	5.75×10^{-6}	3.05x10-1		TSS1500		unclassified
cg10608731	chr6	32155079	PBX2	-0.087 (0.019)	5.83×10^{-6}	5.32x10-2		Body		
cg00370968	chr1	113749386		-0.235 (0.052)	5.86×10^{-6}	9.01x10-1				
cg25362050	chr17	38470900	RARA	-0.107 (0.024)	7.64×10^{-6}	6.40x10-2		5'UTR	x	unclassified
cg21228424	chr6	42678314	PRPH2	0.446 (0.100)	7.67×10^{-6}	9.09x10-1		Body	x	
cg21322248	chr15	77289047	PSTPIP1	-0.412 (0.092)	7.85×10^{-6}	9.74x10-2		Body	x	promoter associated
cg01438090	chr11	30502936	MPPED2	-0.348 (0.078)	8.07×10^{-6}	1.92x10-1		Body	x	
cg16932065	chr22	31612458	LIMK2	-0.150 (0.034)	8.65×10^{-6}	2.33x10-1		Body		
cg03922748	chr2	220142903	DNAJB2	-0.529 (0.119)	8.78×10^{-6}	4.53x10-2		TSS1500	x	

cg19656633	chr5	180074582	FLT4	-0.196 (0.044)	9.54 x10^{-6}	2.64x10-1		Body		
cg26278214	chr6	30173616	TRIM26	-0.268 (0.061)	9.84 x10^{-6}	2.27x10-1		5'UTR		
cg08884752	chr1	2162001	SKI	-0.158 (0.036)	1.04 x10^{-5}	6.30x10-2		Body		
cg14035553	chr1	21029443	KIF17	-0.172 (0.039)	1.06 x10^{-5}	3.81x10-1		Body		
**cg06207201**	**chr16**	**50701535**	**SNX20**	-0.142 (0.032)	1.08 x10^{-5}	3.04x10-1	4.2x10-9	3'UTR		promoter associated
cg06100161	chr15	60987894	RORA	-0.350 (0.080)	1.09 x10^{-5}	7.08x10-2		Body	x	
cg17948136	chr19	23257319		-0.363 (0.083)	1.14 x10^{-5}	3.80x10-1				
cg17010968	chr15	31619006	KLF13	-0.375 (0.086)	1.16 x10^{-5}	8.07x10-1		TSS200		promoter associated
cg12389356	chr4	166382661	CPE	-0.286 (0.065)	1.23 x10^{-5}	9.27x10-1		Body	x	unclassified/cell type specific
cg07543711	chr2	30482168	LBH	-0.400 (0.092)	1.24 x10^{-5}	1.86x10-1		3'UTR	x	
**cg06192883**	**chr15**	**52554171**	**MYO5C**	0.186 (0.043)	1.31 x10^{-5}	3.20x10-3	3.5x10-6	Body	x	unclassified
cg02878284	chr6	32825701	PSMB9	-0.158 (0.036)	1.32 x10^{-5}	2.65x10-1		Body		
cg06096184	chr3	66549732	LRIG1	-0.185 (0.043)	1.32 x10^{-5}	1.32x10-1		Body		
cg17972213	chr1	101704898	S1PR1	-0.163 (0.038)	1.32 x10^{-5}	1.30x10-1		Body		promoter associated

CpG sites in bold correspond to sites identified at significant significance by Wahl et al. [1]. *P-values identified for differentially methylated CpG sites by Al Mufah [2] and of the Europeans within the discovery cohort of Wahl et al. [1] (P**).

Supplementary table 2. Obesity susceptibility genes identified by GWAS that coincide with methylated differentiated loci with P<0.05.

CpG	Effect	SE	P[1]	Chr	Pos	No of diff. sites	Gene
cg17010968	-0.38	0.09	1.16×10^{-5}	chr15	31619006	3	KLF13
cg27347003	0.46	0.11	2.87×10^{-5}	chr16	28915665	4	ATP2A1
cg06153925	0.29	0.07	7.00×10^{-5}	chr17	78755379	24	RPTOR
cg19391675	-0.28	0.08	3.68×10^{-4}	chr6	40345594	1	TDRG1
cg00000924	-0.08	0.02	3.72×10^{-4}	chr11	2720463	26	KCNQ1
cg14886990	-0.16	0.05	4.35×10^{-4}	chr3	52563633	3	NT5DC2
cg27508021	-1.42	0.41	4.57×10^{-4}	chr10	104846094	1	NT5C2
cg18128550	-0.12	0.03	4.75×10^{-4}	chr11	64322146	3	SLC22A11
cg25412453	-0.14	0.04	4.80×10^{-4}	chr1	155006219	1	DCST2
cg06002947	-0.26	0.08	6.78×10^{-4}	chr16	28936713	5	RABEP2
cg05316006	2.52	0.76	8.55×10^{-4}	chr10	114879619	3	TCF7L2
cg16319535	0.50	0.15	0.001	chr5	153599583	5	GALNT10
cg09645291	0.52	0.17	0.002	chr10	79036209	9	KCNMA1
cg18669654	-0.24	0.08	0.002	chr11	63803839	6	MACROD1
cg00534468	0.19	0.06	0.002	chr16	81579629	5	CMIP
cg03866306	-0.56	0.18	0.002	chr13	108039519	3	FAM155A
cg27203924	-0.28	0.09	0.002	chr3	64627542	2	ADAMTS9
cg09584785	-0.35	0.11	0.002	chr1	162333354	5	NOS1AP
cg19353949	0.22	0.07	0.003	chr1	145726979	1	PDZK1
cg09984054	0.98	0.33	0.003	chr8	9634777	1	TNKS
cg22171142	-0.17	0.06	0.003	chr16	3627546	3	NLRC3
cg14585700	-0.19	0.06	0.003	chr9	37027605	5	PAX5
cg03505501	0.22	0.08	0.003	chr11	115371789	6	CADM1
cg14728071	0.17	0.06	0.004	chr10	22030034	1	MLLT10
cg27182923	-0.09	0.03	0.004	chr3	123129387	2	ADCY5
cg07700644	-0.11	0.04	0.004	chr11	116663804	1	APOA5
cg24253904	-0.16	0.05	0.004	chr1	201644923	7	NAV1
cg10596609	-0.22	0.08	0.004	chr2	234525618	1	UGT1A8
cg02578327	-0.18	0.06	0.004	chr2	668370	1	TMEM18
cg15722404	-0.15	0.05	0.004	chr3	12329242	1	PPARG
cg06671139	-0.10	0.04	0.004	chr15	73075935	2	ADPGK
cg10689175	-0.31	0.11	0.004	chr6	34625341	2	C6orf106
cg07398791	-0.12	0.04	0.004	chr5	118676053	4	TNFAIP8
cg20308684	-0.44	0.15	0.005	chr15	67000898	5	SMAD6
cg22727572	-0.64	0.23	0.005	chr2	63272334	1	EHBP1
cg11399254	-0.13	0.05	0.005	chr1	47694517	2	TAL1
cg24142603	-0.34	0.12	0.005	chr8	72753888	1	MSC
cg12905410	-0.18	0.06	0.006	chr2	27805706	1	ZNF512
cg08962038	0.36	0.13	0.006	chr10	88022859	4	GRID1
ch.11.983092R	-4.58	1.88	0.015	chr11	47300267	3	MADD
cg13900542	-0.19	0.08	0.015	chr1	74888995	1	TNNI3K
cg09700598	-0.13	0.05	0.015	chr11	72396376	2	ARAP1
cg08986416	-0.14	0.06	0.015	chr15	51914746	2	DMXL2
cg12815918	-0.18	0.07	0.016	chr13	28498544	2	PDX1
cg00036258	-0.19	0.08	0.016	chr16	4029218	3	ADCY9
cg16115588	-0.16	0.07	0.017	chr12	50297588	3	FAIM2
cg08847132	0.29	0.12	0.017	chr5	173315876	1	CPEB4
cg02111433	0.18	0.08	0.017	chr16	53947953	3	FTO
cg13696409	0.33	0.14	0.019	chr9	73030641	2	KLF9
cg02414034	-0.14	0.06	0.019	chr17	5185283	1	RABEP1
cg25593948	0.11	0.05	0.019	chr6	50786670	4	TFAP2B
cg26020878	0.19	0.08	0.019	chr1	172352681	2	DNM3
cg07803284	0.26	0.11	0.020	chr3	185812471	1	ETV5
cg21010031	0.31	0.13	0.020	chr14	79181274	1	NRXN3
cg17405178	0.31	0.13	0.020	chr6	20572608	1	CDKAL1
cg17971251	-0.22	0.09	0.020	chr1	177907297	1	SEC16B
cg06090362	-0.09	0.04	0.020	chr3	140396963	2	TRIM42
cg13735704	-0.20	0.09	0.021	chr4	103262782	1	SLC39A8
cg00498434	-0.12	0.05	0.022	chr2	212917701	1	ERBB4
cg12416929	-0.07	0.03	0.022	chr6	108882205	3	FOXO3
cg17101778	-0.15	0.07	0.022	chr16	28875557	1	SH2B1
cg20557037	0.26	0.11	0.023	chr2	165698099	1	COBLL1
cg25448062	-0.17	0.08	0.023	chr11	61580118	1	FADS1
cg00000714	-0.11	0.05	0.023	chr19	54695678	1	TSEN34
cg25841625	-0.19	0.08	0.024	chr4	77119588	2	SCARB2
cg03471150	-0.07	0.03	0.024	chr1	201797198	1	IPO9
cg24037647	-0.17	0.08	0.024	chr1	49175771	2	AGBL4
cg05907649	0.17	0.07	0.024	chr15	56519080	1	RFX7
cg21885638	-0.09	0.04	0.025	chr3	129306552	3	PLXND1
cg23026345	-0.15	0.07	0.025	chr6	40400462	3	LRFN2
cg09922770	0.17	0.08	0.026	chr19	46196180	1	QPCTL
cg26534213	-0.28	0.13	0.026	chr1	78149566	2	ZZZ3
cg26931308	0.17	0.08	0.026	chr10	22292816	2	DNAJC1
cg14544180	-0.13	0.06	0.027	chr19	18383319	1	KIAA1683
cg01305421	0.12	0.05	0.027	chr12	102874286	1	IGF1
cg15001372	-0.11	0.05	0.027	chr7	44229892	1	GCK
cg04348872	-0.17	0.08	0.027	chr2	25141696	2	ADCY3
cg24065044	-0.53	0.24	0.028	chr11	27723409	3	BDNF

cg02234120	-0.20	0.07	0.006	chr2	234626351	1	UGT1A10
cg00986598	-1.30	0.47	0.006	chr3	129369724	2	TMCC1
cg10904070	-0.12	0.04	0.006	chr18	3012017	3	LPIN2
cg19977866	-0.61	0.22	0.006	chr11	92705264	1	MTNR1B
cg00084184	-0.19	0.07	0.007	chr4	10019009	2	SLC2A9
cg02075822	-0.10	0.04	0.007	chr7	75365585	2	HIP1
cg25570453	-0.20	0.07	0.007	chr7	14834316	1	DGKB
cg22820316	0.14	0.05	0.008	chr1	119532736	4	TBX15
cg07752026	-0.13	0.05	0.008	chr19	3786483	3	MATK
cg25059899	0.12	0.05	0.008	chr18	60904328	4	BCL2
cg23931836	-0.25	0.10	0.009	chr19	47615988	2	ZC3H4
cg05805445	0.09	0.04	0.009	chr4	124225943	2	SPATA5
cg16969872	-0.15	0.06	0.009	chr13	79968324	3	RBM26
cg14455516	0.19	0.07	0.010	chr2	27165781	1	DPYSL5
cg02793733	-0.17	0.07	0.010	chr2	26928245	2	KCNK3
cg13979277	-0.19	0.07	0.010	chr9	129388281	3	LMX1B
cg12157519	-0.08	0.03	0.011	chr9	72874251	2	SMC5
cg01351783	-0.25	0.10	0.011	chr6	7220085	6	RREB1
cg20272884	-0.19	0.08	0.011	chr8	23712762	1	STC1
cg17361641	-0.25	0.10	0.011	chr19	18391832	1	JUND
cg13930468	-0.15	0.06	0.012	chr4	18022372	2	LCORL
cg20434926	0.29	0.12	0.013	chr19	46181274	2	GIPR
cg12266551	-0.18	0.07	0.013	chr19	45394624	2	TOMM40
cg23714707	-0.18	0.07	0.013	chr17	17418691	1	PEMT
cg13948824	0.41	0.17	0.014	chr12	26492184	1	ITPR2
cg08781803	-0.13	0.05	0.014	chr15	67834857	2	MAP2K5
cg15505219	0.33	0.13	0.014	chr14	61858317	2	PRKCH
cg05516617	-0.10	0.04	0.014	chr7	27221689	1	HOXA11
cg02397182	-0.28	0.11	0.014	chr6	43739819	1	VEGFA
cg09046979	-0.18	0.08	0.028	chr16	28333134	2	SBK1
cg03009080	0.38	0.17	0.028	chr13	28024744	1	MTIF3
cg07356753	-0.09	0.04	0.029	chr3	81810745	1	GBE1
cg27338487	-0.13	0.06	0.030	chr18	56887404	1	GRP
cg24305156	-0.06	0.03	0.030	chr11	45871936	2	CRY2
cg10500512	-0.16	0.08	0.031	chr20	22564041	2	FOXA2
cg17183991	-0.26	0.12	0.032	chr13	96743170	1	HS6ST3
cg04196862	-0.10	0.05	0.032	chr17	46669455	1	HOXB5
cg22215728	-0.21	0.10	0.032	chr3	61236652	2	FHIT
cg22534898	-0.14	0.06	0.033	chr3	52865592	1	ITIH4
cg12398332	-0.33	0.16	0.033	chr16	19895651	1	GPRC5B
cg20273358	-0.19	0.09	0.033	chr20	56100118	1	CTCFL
cg25499897	0.15	0.07	0.033	chr12	122907396	2	CLIP1
cg13096436	-1.09	0.51	0.033	chr18	21083456	1	C18orf8
cg00498289	-0.09	0.04	0.035	chr2	27719787	1	GCKR
cg23922739	0.12	0.06	0.036	chr12	54338743	2	HOXC13
cg01263075	-0.17	0.08	0.036	chr4	89080199	1	ABCG2
cg11404544	-0.11	0.05	0.036	chr1	50571816	1	ELAVL4
cg22759265	-0.32	0.15	0.036	chr2	27998164	2	MRPL33
cg16276290	0.22	0.11	0.037	chr19	34287540	2	KCTD15
cg08314679	0.28	0.14	0.038	chr10	102295474	1	HIF1AN
cg18688704	-0.11	0.05	0.039	chr4	157889520	2	PDGFC
cg08802358	-0.13	0.06	0.040	chr5	176524797	3	FGFR4
cg25207224	-0.05	0.03	0.040	chr6	34211189	1	HMGA1
cg15892497	-0.17	0.08	0.041	chr1	145715719	1	CD160
cg24913935	0.25	0.12	0.041	chr11	116643957	1	BUD13
cg26927232	-1.22	0.60	0.041	chr17	2135631	1	SMG6
cg18040826	0.50	0.25	0.043	chr3	156543194	1	LEKR1

[1] The p-values listed are the lowest identified of the differential sites linked to each GWAS associated locus.

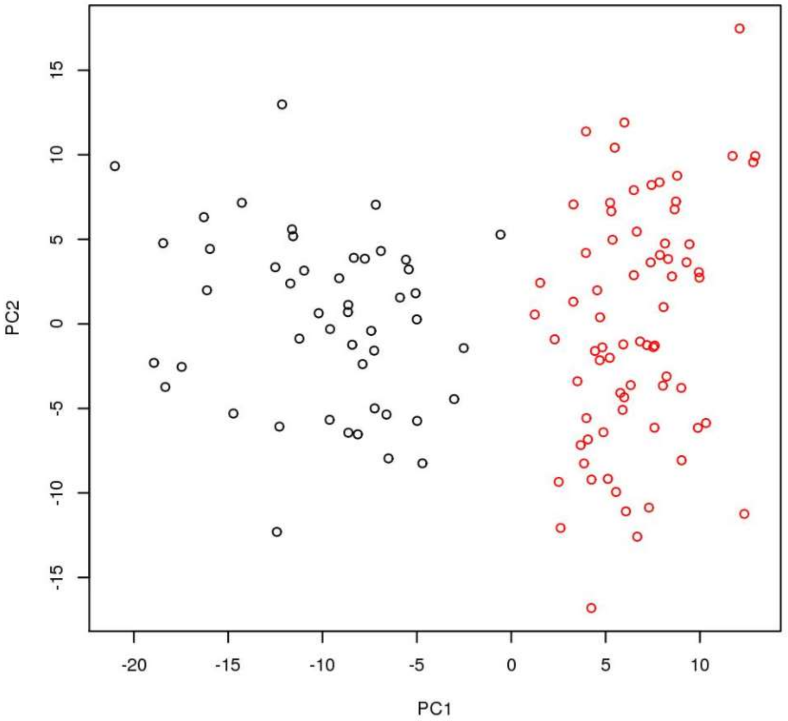

Supplementary figure 1. Illustration of batch effect using principal component analysis.

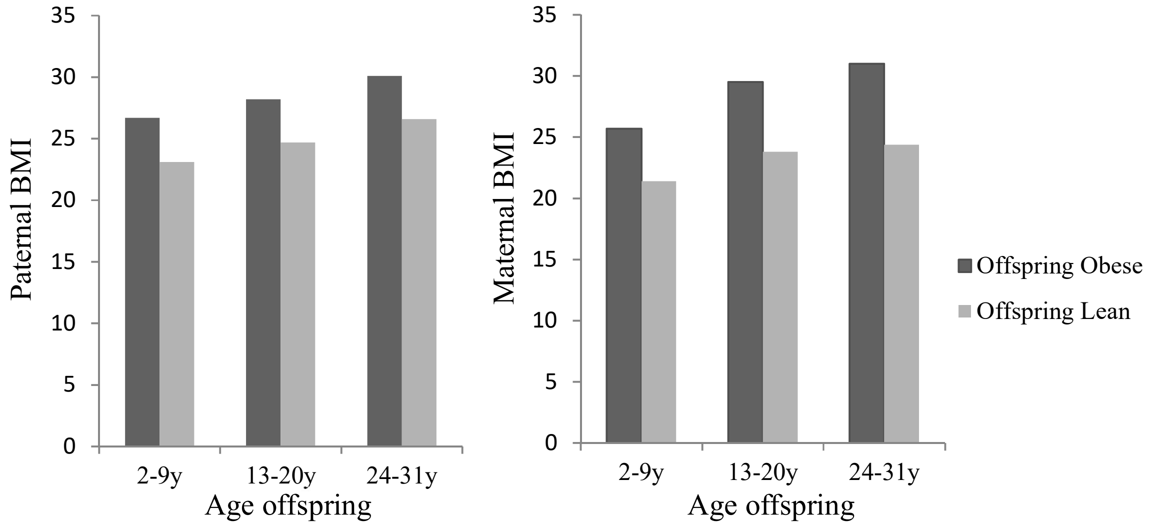

Supplementary figure 2. Weight data (body mass index) from parents of the 120 study participants who had also participated in the HUNT Study at three different time points (1984-86, 1995-97 and 2006-08) within a time span of 20 years.

References

1. Wahl S, Drong A, Lehne B, Loh M, Scott WR, Kunze S, et al. Epigenome-wide association study of body mass index, and the adverse outcomes of adiposity. Nature. 2017;541(7635):81-6. Epub 2016/12/22. doi: 10.1038/nature20784. PubMed PMID: 28002404.
2. Al Muftah WA, Al-Shafai M, Zaghlool SB, Visconti A, Tsai PC, Kumar P, et al. Epigenetic associations of type 2 diabetes and BMI in an Arab population. Clinical epigenetics. 2016;8:13. Epub 2016/01/30. doi: 10.1186/s13148-016-0177-6. PubMed PMID: 26823690; PubMed Central PMCID: PMCPmc4730771.
